# Infrared Spectroscopy as a Tool to Study the Antioxidant Activity of Polyphenolic Compounds in Isolated Rat Enterocytes

**DOI:** 10.1155/2016/9245150

**Published:** 2016-04-26

**Authors:** Guillermo Barraza-Garza, Hiram Castillo-Michel, Laura A. de la Rosa, Alejandro Martinez-Martinez, Jorge A. Pérez-León, Marine Cotte, Emilio Alvarez-Parrilla

**Affiliations:** ^1^Departamento de Ciencias Químico Biológicas, Instituto de Ciencias Biomédicas, Universidad Autónoma de Ciudad Juárez, 32310 Ciudad Juárez, CHIH, Mexico; ^2^X-ray and Infrared Microspectroscopy Beamline ID21, European Synchrotron Radiation Facility, BP 220, 38043 Grenoble Cedex, France

## Abstract

The protective effect of different polyphenols, catechin (Cat), quercetin (Qc) (flavonoids), gallic acid (GA), caffeic acid (CfA), chlorogenic acid (ChA) (phenolic acids), and capsaicin (Cap), against H_2_O_2_-induced oxidative stress was evaluated in rat enterocytes using Attenuated Total Reflectance-Fourier Transform Infrared (ATR-FTIR) Spectroscopy and Fourier Transform Infrared Microspectroscopy (FTIRM), and results were compared to standard lipid peroxidation techniques: conjugated dienes (CD) and Thiobarbituric Acid Reactive Substances (TBARS). Analysis of ATR-FTIR and FTIRM spectral data allowed the simultaneous evaluation of the effects of H_2_O_2_ and polyphenols on lipid and protein oxidation. All polyphenols showed a protective effect against H_2_O_2_-induced oxidative stress in enterocytes, when administered before or after H_2_O_2_. Cat and capsaicin showed the highest protective effect, while phenolic acids had weaker effects and Qc presented a mild prooxidative effect (IR spectral profile of biomolecules between control and H_2_O_2_-treated cells) according to FTIR analyses. These results demonstrated the viability to use infrared spectroscopy to evaluate the oxidant and antioxidant effect of molecules in cell systems assays.

## 1. Introduction

Oxidative stress is caused by an overproduction of reactive oxygen species (ROS); this state is known to be involved in the development of numerous diseases including cardiovascular and neurodegenerative disease, among others [1]. Polyphenols are natural antioxidant compounds ubiquitous in plant foods, known for their ROS-scavenging activity in* in vitro* systems and their protective effect against oxidative stress-related diseases. However, due to their low bioavailability, there is controversy about their mechanism of action, and direct* in vivo* ROS-scavenging activity is questionable [1, 2]. An indirect antioxidant effect of polyphenols has been recently proposed, through the upregulation of the cellular antioxidant defense system (phase 2 enzymes), via activation of the Nrf2-Keap1 pathway [3–6]. Polyphenols may be autooxidized with the formation of H_2_O_2_ and quinoidal products and both chemical species may participate in electrophilic conjugation reactions with cysteine residues of the Nrf2-Keap1 complex, allowing the translocation of Nrf2 and thus starting the pathway [6–8]. In this scenario, autooxidation products of polyphenols are actually mild prooxidants. Alternatively, polyphenols may activate the Nrf2 pathway by mechanisms apparently independent of their electrophilic properties [6–8].

**Figure 1:**
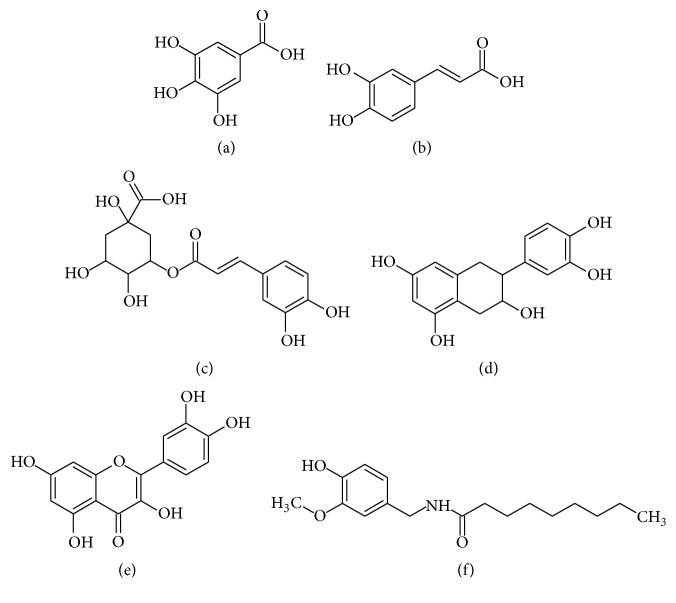
Molecular structure of the polyphenolic compounds used in this work. (a) Gallic acid (GA). (b) Caffeic acid (CfA). (c) Chlorogenic acid (ChA). (d) Catechin (Cat). (e) Quercetin (Qc). (f) Capsaicin (Cap).

Enterocytes are a good model to study the mechanisms by which polyphenols exert their antioxidant activity, because they are the first cells to interact with polyphenols during the absorption process and, consequently, they are exposed to the highest concentrations of polyphenols among all cell types in the body. However, there is little information about what happens in these cells when they are under oxidative stress and in presence of polyphenols [6]. Attenuated Total Reflectance-Fourier Transform Infrared (ATR-FTIR) Spectroscopy and synchrotron radiation-based Fourier Transform Infrared Microspectroscopy (FTIRM) have been recently used to evaluate oxidative stress, like photo-oxidative damage, X-ray radiation, and H_2_O_2_ stressors in different types of cells like fibroblast, carcinogenic cells, and plasmatic cells, being able to correlate the shifts in the infrared signals with lipid and protein peroxidation [9–11]. Previously, our group used FTIRM to evaluate lymphocytes of rats under psychological stress, observing lipid peroxidation in the cells of stressed individuals [12]. This prompted us to investigate the antioxidant mechanisms of different types of polyphenols in enterocytes exposed to oxidative stress. In this work, we report the protective effect of several polyphenolic compounds which include three phenolic acids, gallic acid (GA), caffeic acid (CfA), and chlorogenic acid (ChA), two flavonoid compounds, catechin (Cat) and quercetin (Qc), and one vanilloid, capsaicin (Cap). The structures of these polyphenolic compounds can be seen in Figure 1, where we can see the structural differences among them. These polyphenolic compounds were tested against oxygen peroxide-induced oxidative stress by means of infrared spectroscopy and these results were compared with those obtained by standard techniques such as conjugated dienes (CD) and Thiobarbituric Acid Reactive Substances (TBARS). This study demonstrates the applicability of FTIR to investigate the biochemical status of enterocytes under induced oxidative stress and their response to polyphenols.

## 2. Materials and Methods

### 2.1. Ethics Statement

Experiments were approved by the Bioethics Committee of the Universidad Autónoma de Ciudad Juárez (Autonomous University of Ciudad Juarez, UACJ). Animals were handled and cared according to the NIH care and use of laboratory animals [13].

### 2.2. Chemicals and Reagents

All polyphenolic compounds (catechin [Cat], quercetin [Qc], gallic acid [GA], caffeic acid [CfA], chlorogenic acid [ChA], and capsaicin [Cap]), chloroform, cyclohexane, H_2_O_2_, thiobarbituric acid, sodium bicarbonate, trypan blue and penicillin-streptomycin, trichloroacetic acid, hydrochloric acid, and tetramethoxypropane (TMP) were purchased from Sigma-Aldrich (Mexico City, Mexico). Pentobarbital and metronidazole were purchased from PiSa Laboratories (Guadalajara, Mexico). Methanol was purchased from Hycel (Jalisco, Mexico). Dulbecco's modified Eagle's medium (DMEM), fetal bovine serum (FBS), and Tryple Express*™* were purchased from Gibco (Gaithersburg, Maryland, USA). Isolation medium consisted of DMEM adjusted to pH 7.4 with bicarbonate, containing 10% of FBS 1% of penicillin-streptomycin. TBARS solution was made with trichloroacetic acid 15%, thiobarbituric acid 0.4%, and hydrochloric acid 2.5%.

### 2.3. Cell Isolation

Enterocytes were isolated from the small intestine of one-month-old Sprague-Dawley rats following the method of Chougule et al., [14] with modifications. Rats were euthanized with pentobarbital (100 mg/kg) via intraperitoneal injection. The duodenum and jejunum were dissected by cutting a section of 15–20 cm starting from the pylorus. The extracted intestine was placed in ice chilled NaCl 0.15 M prepared in phosphate buffer (0.01 M) pH 7.4 (PBS) containing penicillin-streptomycin 1% and metronidazole 5%. Afterwards, the intestine was cleaned, sectioned, and washed in PBS with antibiotics (penicillin-streptomycin 1% and metronidazole 5%). The intestine sections were exposed to Tryple Express 1x with gentle agitation for 15 min and vortexed for 30 seconds. Intestine sections were disposed and the cell suspension was centrifuged for 10 min at 201 ×g. The supernatant was discarded and the cells were suspended in 1 mL of DMEM with antibiotics for further treatment. A sample of 50 *μ*L of the cell suspension was mixed (1 : 1) with 0.4% trypan blue and used for cell counting and viability in a Neubauer chamber, viewed with an upright microscope (Leica CME Leica Miscrosystems, Wetzlar, Germany) [14, 15].

### 2.4. Polyphenol Treatments

Between 1,000,000 and 1,500,000 cells/mL were used per treatment. Six polyphenols were used in this study: Cfa, ChA, GA, Cat, Cap, and Qc at a final concentration of 100 *μ*M. H_2_O_2_ at a final concentration of 500 *μ*M was used to induce oxidative stress. Three different treatments were used for each polyphenol: (i) only polyphenols added to the medium, (ii) polyphenols added to the medium before H_2_O_2_, and (iii) polyphenols added to the medium after H_2_O_2_. Cells were exposed to H_2_O_2_ and polyphenols for 30 min each. Control cells or those treated with just one compound were first preincubated for 30 min with control medium, washed with PBS (pH 7.4), centrifuged at 95 ×g for 5 min, and then treated with the corresponding compound for another 30 min. Cells treated with polyphenols before or after H_2_O_2_ were incubated with the first compound for 30 min, washed, centrifuged, and incubated with the second compound for another 30 min. After their treatments, cells were centrifuged and the supernatant was discarded. Then, cells were washed twice in PBS (pH 7.4), centrifuged at 95 ×g for 5 min, and then suspended in different solutions depending on the following analyses. For FTIR and FTIRM experiments, cells were fixed in paraformaldehyde 4% in PBS (PFA-PBS) for 20 min. Fixed cells were centrifuged for 10 min at 95 ×g, supernatant was discarded, and the cells were washed in deionized water and finally suspended in 200 *μ*L of water for infrared spectroscopy analysis [12].

### 2.5. Fourier Transform Infrared Microspectroscopy (FTIRM) and Attenuated Total Reflectance-Fourier Transform Infrared (ATR-FTIR) Spectroscopy

Fixed cells analyses in FTIR-ATR were carried out in a Thermo Nicolet Nexus 670 FTIR spectrometer (Thermo Scientific, Madison, WT, USA). Fixed cells (300,000 to 350,000 cells) were dried directly in the Ge crystal of the Attenuated Total Reflectance (ATR) for 20 to 30 min. Spectra were recorded in the range of 4000 to 800 cm^−1^ with a maximum resolution of 6 cm^−1^, and 150 scans per spectrum were collected. Five spectra were recorded for each treatment population.

Fixed cells were observed using the FTIRM end station in the line ID-21 at the European Synchrotron Radiation Facility (ESRF) in Grenoble, France, using a Thermo Nicolet Continuum (Thermo Scientific, Madison, WT, USA) microscope coupled to a Thermo Nicolet Nexus FTIR spectrometer (Thermo Scientific, Madison, WT, USA). The IR microscope was equipped with a 32x objective, a motorized sample stage, and a liquid nitrogen-cooled 50 *μ*m mercury cadmium telluride detector. Fixed cells were mounted in BaF_2_ windows of 1 mm height. Spectra were recorded over the range of 4000 to 850 cm^−1^, the spectral resolution was set to 6 cm^−1^, and 120 scans per spectrum were collected. Spectra of 30 individual cells were recorded for each treatment.

### 2.6. Infrared Spectra Analysis

Infrared spectra were analyzed using Unscrambler X software (CAMO Software, Norway). Raw spectra were preprocessed using first vector normalization and then a second derivative using Savitsky-Golay of second polynomial order with 21 smoothing points [16, 17]. Second derivative spectra were used for the calculation of ratios of IR signals at different bands. Effects of oxidative stress have been related to the displacement/reduction of some IR bands; for lipid oxidation, the analyzed bands are 1740 cm^−1^/2960 cm^−1^, for lipid saturation, 2920 cm^−1^/2960 cm^−1^, and lipid desaturation, 3012 cm^−1^/2960 cm^−1^. Meanwhile, for proteins aggregation bands ratios 1630 cm^−1^/1650 cm^−1^ have been analyzed [18–20]. The use of the second derivative has been previously reported and is useful for the study of masked bands [18, 21, 22].

IR spectra were also analyzed by principal component analysis (PCA), which has been previously used to compare cells under oxidative stress and other treatments [17, 23]. Spectra were analyzed in full range from 4000 to 900 cm^−1^ and in two particular sections: 1900 to 900 cm^−1^ (related to proteins and nucleic acids) and 3200 to 2800 cm^−1^ (related to lipids). Both score plots and loading plots were obtained by PCA analysis.

### 2.7. Determination of Lipid Peroxidation by Conjugated Dienes (CD)

Conjugated dienes were measured according to Devasagayam et al., [24] with slight modifications. After treatment, a pellet composed of 300,000 cells was suspended in PBS and immediately frozen at −20°C for 2-3 days until analysis. Cells were thawed, washed with PBS (pH 7.4), and centrifuged for 5 min at 9500 ×g. Pellet was resuspended in 300 *μ*L of methanol for lipid extraction. Cells were lysed by sonication for 1 min at 0°C using a Sonic Dismembrator Model 100 homogenizator (Fisher Scientific, Pittsburgh, PA, USA) and then vortexed vigorously for 1 min at 68 ×g and 300 *μ*L of chloroform was added. The mixture was vortexed for 2 min at 68 ×g; 600 *μ*L of chloroform was added and vortexed again for another 2 min at 68 ×g. The mixture was centrifuged at 4°C for 10 min at 4650 ×g and the lower chloroform phase was collected and dried with a nitrogen flux. Lipids were then dissolved in 500 *μ*L of cyclohexane, vortexed for 30 seconds at 68 ×g, and immediately measured with a UV-spectrophotometer at 233 nm (Thermo Scientific, Madison, WT, USA) using cyclohexane as blank. Absorbance was converted to conjugated dienes concentration using an extinction coefficient of 27000 M^−1^ cm^−1^ and the results were expressed as concentration (*μ*M) of conjugated dienes per million of cells.

### 2.8. Determination of Thiobarbituric Acid Reactive Substances (TBARS)

TBARS were measured according to Devasagayam et al., [24] with modifications [25]. Cell samples were frozen as described. Cells (300,000) were thawed and then washed with PBS (pH 7.4) and centrifuged for 5 min at 9500 ×g. Pellet was suspended in 300 *μ*L of PBS; cells were lysed by sonication for 1 min in ice bath using a Sonic Dismembrator Model 100 homogenizator (Fisher Scientific, Pittsburgh, PA, USA) and then vortexed vigorously for 1 min at 68 ×g. Samples were then centrifuged for 5 min at 9500 ×g and resuspended in 200 *μ*L of PBS. A calibration curve was prepared with tetramethoxypropane (TMP) with concentrations ranging from 1.25 to 20 *μ*M, which are equivalent to the same concentrations of MDA, a peroxidation byproduct. Samples and calibration standards were mixed with 400 *μ*L of TBA reagent (20% TCA, 0.5% TBA, and 2.5 N HCl) and the mixture was heated for 45 min in a boiling water bath. After cooling, the solution was centrifuged at 380 ×g for 10 min and the absorbance of the supernatant was measured at 532 nm using the reaction mixture as a blank. Results were expressed as concentration (*μ*M) of MDA per million of cells.

### 2.9. Statistical Analysis

Results obtained with CD and TBARS were analyzed with a one-way analysis of variance (ANOVA). When ANOVA showed a significant difference, Tukey's* post hoc* test was applied. Statistical significance was regarded as *P* < 0.05. Multivariate analysis was made by PCA as described in the previous subsection, using Unscrambler X software (CAMO Software, Norway).

## 3. Results and Discussion

**Figure 2:**
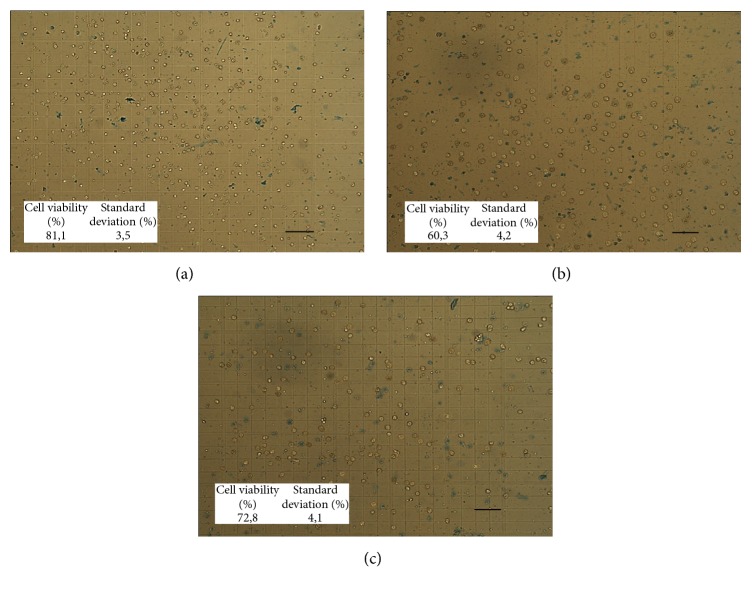
Cell viability after treatments. Enterocytic cells after isolation from the rat intestine and exposed to their different treatments. (a) Control cell after 1 hour in DMEM plus antibiotics. (b) Cells exposed for 30 min to DMEM medium with 0.5 mM of H_2_O_2_ first and then transferred to base DMEM medium, for 30 min each. (c) Cells exposed to DMEM-0.1 mM gallic acid first for 30 min and afterwards transferred to H_2_O_2_ for 30 min for each treatment. Measurements of viable cells were made by using 0.1% trypan blue exclusion assay with a Neubauer chamber average of percentage of viability reported in Results. Cell viability and SD noted for each treatment. Scale bar 40 *μ*m.

Viability tests were performed with trypan blue in freshly isolated enterocytes and after applying all treatments. Immediately after isolation, cell viability was around 80% and decreased to 75% and 72% after 1 hour for the control treatment or with the addition of polyphenols (see Figure 2(a) for visualization of cell morphology). These viability values are similar to those reported by others (between 50% and 90%) for the isolation of intestinal cells [14, 26]. Cells treated with H_2_O_2_ for 1 hour showed 60.3% viability (Figure 2(b)) and all H_2_O_2_-phenol and phenol-H_2_O_2_ treated cells showed viabilities ranging from 66% to 79% (Figure 2(c)). By obtaining these data, we can assure that the cells used in infrared spectroscopy and the other biochemical tests were mostly viable cells.

### 3.1. Evaluation of Oxidative Stress and Antioxidant Activity by Analysis of Infrared Spectral Data

**Figure 3:**
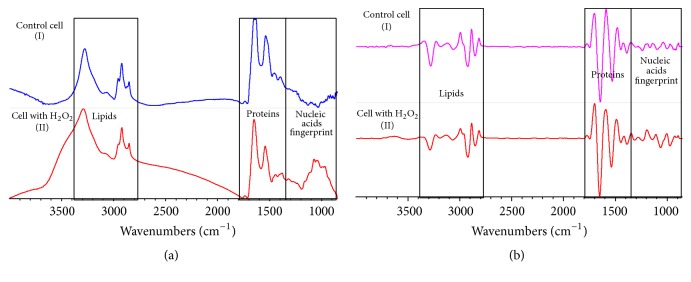
Infrared spectra obtained using FTIRM and processed with a second derivative. (a) Average spectrums obtained with the control treatment (DMEM) during 1 hr (I) and H_2_O_2_-treated cells (DMEM added with 0.5 mM of H_2_O_2_) 30 min (II), spectra were obtained with a spectral resolution set to 6 cm^−1^. (b) Average spectra processed with second derivative using Savitsky-Golay with 21 smoothing points and a second polynomial order. Control treatment cells (DMEM) during 1 hr (I) and H_2_O_2_-treated cells (DMEM added with 0.5 mM of H_2_O_2_) for 30 min (II). The differences seen in these treatments were analyzed by the PCA analysis to classify them and to know the most important variations in the dataset. There are two major zones of interest: proteins zone (1720–1470 cm^−1^) and lipids zone (3200–2800 cm^−1^).

**Figure 4:**
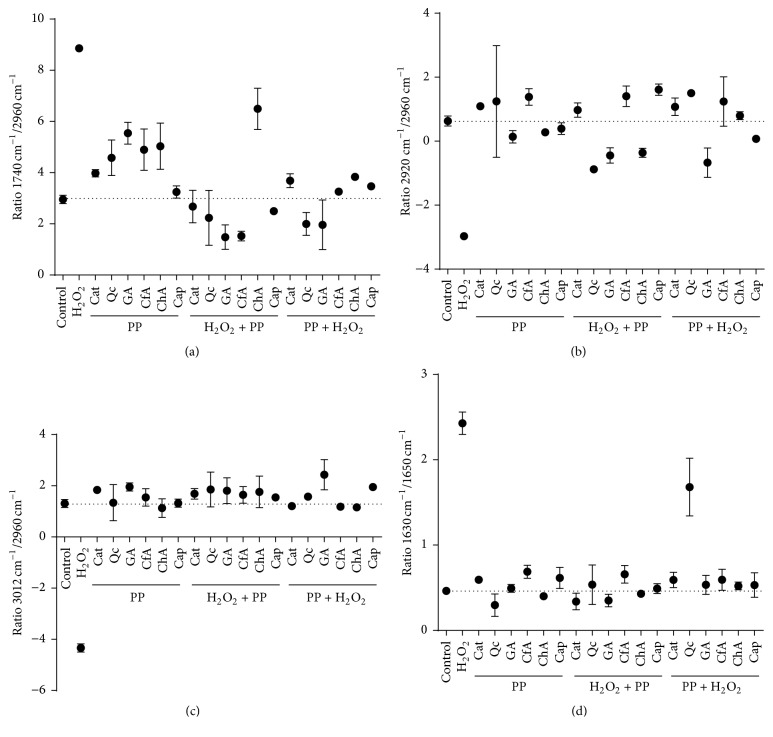
Ratios between chemical bonds related to the lipid peroxidation observed with the FTIR spectra data. Those ratios were as follows: (a) the ratio assigned to 1740 cm^−1^/2960 cm^−1^ is related to lipid oxidation, (b) the ratio assigned to 2920 cm^−1^/2960 cm^−1^ is related to lipid saturation, (c) the ratio assigned to 3012 cm^−1^/2960 cm^−1^ is related to lipid desaturation, and (d) the ratio assigned 1630 cm^−1^/1650 cm^−1^ is related to protein aggregation. The dotted line indicates the mean value of the control cells for each ratio; all ratios were compared to the control ratio in each case. PP: polyphenol.

Infrared spectroscopy allowed us to have an IR spectral profile of the biomolecules of cells under oxidative stress and, by comparison with the IR spectral profile of control cells or polyphenol-treated cells, elucidate the changes in different biomolecules (lipids and proteins) in response to polyphenol treatments. In order to confirm the suitability of FTIR spectral data to analyze the H_2_O_2_-induced oxidative stress and antioxidant effect of polyphenolic compounds in rat enterocytes, an experiment was carried out, using six polyphenols (Cap, Cat, CfA, ChA, GA, and Qc). FTIR spectra were measured with an ATR-FTIR spectrometer with globar source of light in populations of 300,000 to 350,000 cells. ATR-FTIR spectral analyses were performed considering two main regions of interest (see Figure 3(a)): aldehyde and amide I and amide II in the protein region (1750–1470 cm^−1^) and CH_2_ and CH_3_ alkyl chains and olefinic bond in the lipid region (3200–2800 cm^−1^). In order to better identify the differences between spectra, they were vector-normalized followed by a second derivative transformation (Figure 3(b)). Second derivative allowed us to observe absorption bands with fine structures that normally are masked in the raw data but that can be important markers of the oxidative status of the cells and can be used for comparative analysis between treatments. In Figure 3(b), absorbance and second derivative representative spectra from control and H_2_O_2_-treated cells are shown. Results indicate that H_2_O_2_-treated cells suffered modifications in their IR spectral profiles. Protective effects of individual polyphenolic compounds were determined by analyzing the ratios between the intensity of FTIR signals associated with lipid and protein oxidation. Those ratios were 1740 cm^−1^/2960 cm^−1^ (lipid oxidation), 2920 cm^−1^/2960 cm^−1^ (lipid saturation), 3012 cm^−1^/2960 cm^−1^ (lipid desaturation), and 1630 cm^−1^/1650 cm^−1^ (protein aggregation). These markers were interpreted by comparing the results of treated cells against those of control cells, in agreement with previous studies that have used these ratios to evaluate oxidative stress in neurons of patients suffering from Alzheimer's disease [18]. For the first ratio, which indicates lipid oxidation (1740 cm^−1^/2960 cm^−1^) related to the increase of aldehyde groups from lipid peroxidation [16, 18], Figure 4(a) shows that H_2_O_2_-treated cells presented the highest value, which was statistically different to control cells. For this marker, the treatments “ChA,” “GA,” “Qc,” and “H_2_O_2_ + ChA” showed higher values than control cells and the other polyphenolic treatments, but lower than H_2_O_2_-treated cells. PCA of these spectra (data not shown) also showed that most polyphenolic treatments protected cells from H_2_O_2_-induced oxidative damage but treatment of cells with polyphenols in the absence of H_2_O_2_ induced a mild prooxidant effect. It has been reported that small polyphenols have a direct action against ROS but also show a prooxidant effect [6]. Both phenolic acids (ChA and GA) showed the lowest antioxidant effect in all the analyzed treatments, indicating that the formation of aldehydes is higher than the capacity of the phenolic acids to exert their antioxidant effect.

For the results of the second ratio (2920 cm^−1^/2960 cm^−1^), which corresponds to lipid saturation, cells exposed only to H_2_O_2_ have lower values than both control and cells treated with polyphenols, indicating a lower level of saturated lipids. The 3012 cm^−1^/2960 cm^−1^ ratio, which indicates lipid desaturation, was also lowest in H_2_O_2_-treated cells (Figures 4(b) and 4(c)). Treatments “GA + H_2_O_2_,” “H_2_O_2_ + ChA,” “H_2_O_2_ + GA,” and “H_2_O_2_ + Qc” had lower values than the control for lipid saturation, while GA + H_2_O_2_ showed the highest value of all polyphenol treatments, for the level of lipid desaturation. These two markers indicated a lower antioxidant effect of phenolic acids in comparison with Cat and Cap, and in intermediate effect of Qc, in agreement with studies that show prooxidant activity of this polyphenol [27, 28]. The fact that bigger molecules showed better results can be related to an indirect action of these compounds through activation of antioxidant enzymes which enables a better response against oxidative stress in the cell [6]. Finally, the ratio 1630 cm^−1^/1650 cm^−1^, related to protein aggregation, was only affected (increased) by the treatment with H_2_O_2_ and Qc + H_2_O_2_; this again indicates a mild prooxidant effect of Qc or a low antioxidant effect in protecting proteins from H_2_O_2_-induced aggregation.

**Figure 5:**
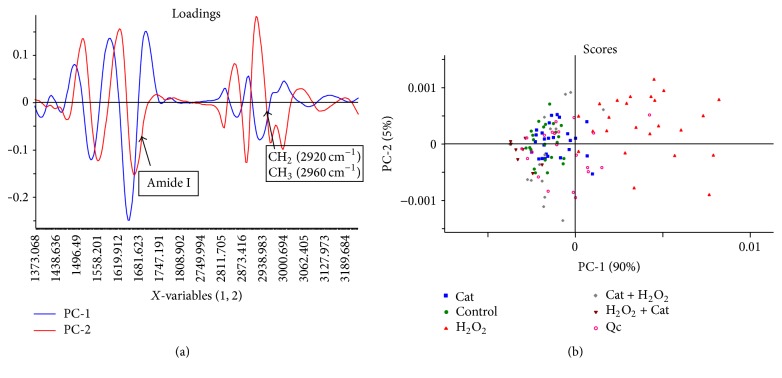
Results obtained with FTIRM infrared spectra data using PCA. (a) Loading plot from the FTIRM samples observed in both protein region (1800–900 cm^−1^) and lipid region (3200–2800 cm^−1^) for PC-1 and PC-2. (b) Score plot from the FTIRM samples in the protein wavenumbers (1900–900 cm^−1^) and the lipid zone of wavenumbers (3200–2800 cm^−1^) for PC-1 and PC-2. The number of samples used per treatment was 30. Main changes are observed as a shift in amide I (1660 cm^−1^) and changes in the relations between CH_2_ (2920 cm^−1^) and CH_3_ (2960 cm^−1^) bonds.

After using ATR-FTIR to analyze oxidative stress in enterocytes, a second experiment was performed using synchrotron radiation-based FTIRM. In this experiment, only Cat and Qc were used to observe if the effects determined using ATR-FTIR were also observed in individual cells using FTIRM. Results from PCA (Figure 5) showed that our data matrix can be reduced to 2 principal components describing 95% of the total variance in the data.  Figure 5(a) shows the loading plot of both regions of interest (proteins and lipids) with PC-1 and PC-2. Changes are observed in the amide I band (1660 cm^−1^) in PC-1, corresponding to the area in which the control cells and the polyphenolic treatments are grouped in the score plot (Figure 5(b)). The shift in amide I is related to the protein aggregation which is seen as a change in the protein structure and this effect is caused by the oxidative stress in cells [18, 20]. Loading plot of the lipid region (Figure 5(a)) showed a change in the intensity of the bands from 2960 cm^−1^ to 2920 cm^−1^, which corresponded to the asymmetric stretch from CH_3_ and CH_2_, respectively, which are linked to the state of saturation of lipids in the cell [16, 29], in agreement with the results of the previously described FTIR experiment.

The score plot (Figure 5(b)) for the two regions of interest showed that cells treated with Cat before and after H_2_O_2_ were grouped closest to control cells, indicating that treatment with this polyphenol maintained an IR spectral profile similar to control cells, due to its protective effect against H_2_O_2_-induced oxidative stress. Cells treated with Qc alone were found in an intermediate zone between samples from control cells and cells under oxidative stress. This can be associated with the known fact that polyphenols may also act as mild prooxidant molecules, an effect that has been documented for Qc. This compound has been reported to show prooxidant and cytotoxic effects in primary splenocytes (LC50 of 150 and 188 *μ*M, resp.) and these effects were blocked by antioxidants and inhibitors of cytochrome P-450 [27]. Cells with H_2_O_2_ are far from the control cells making its own group which is differentiated from the other treatments as an effect of the oxidative stress in enterocytes.

In summary, analysis of FTIR spectra indicated that phenolic acids and Qc showed lower protective effect than Cat and Cap. This could be related mostly to the activation of endogenous antioxidant pathways [30] which are not activated by phenolic acids [6] and to a partially prooxidant effect of Qc [6, 27]. This data also indicates that a stronger antioxidant activity* in vitro* (e.g., due to the presence of three hydroxyl groups in GA) does not necessarily correlate with a higher protective activity* in vivo *[30, 31].

### 3.2. Evaluation of Lipid Oxidation by Conjugated Dienes (CD) and Thiobarbituric Acid Reactive Substances (TBARS)

**Figure 6:**
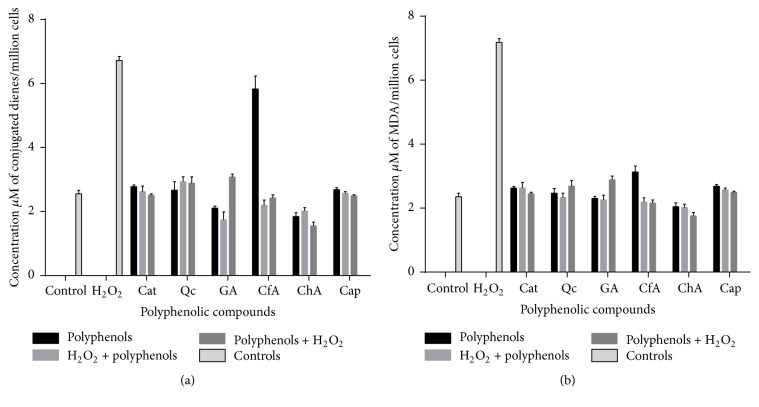
(a) Conjugated dienes (CD) assay. Concentration of CD per million cells observed in the full battery of control and polyphenolic treatments. Values are the mean + SD from six measurements. (b) TBARS assay. Concentration of MDA equivalents per million cells observed in the full battery of control and polyphenolic treatments. Values are the mean + SD from six measurements.

In order to compare the FTIRM and FTIR results with classical biochemical assays, primary and secondary lipid oxidation products were measured by CD and TBARS, respectively.  Figure 6 depicts the CD (a) and TBARS (b) values for all treatments. H_2_O_2_-treated cells showed the highest content of peroxidation products in both assays and all phenolic compounds behaved as antioxidants protecting against H_2_O_2_-induced lipid oxidation. Content of CD in cells treated with Cat, Cap, and Qc, in the presence or absence of H_2_O_2_, remained the same as control values (Figure 6(a)), indicating that these compounds blocked H_2_O_2_-induced CD production. Reduction of CD by polyphenols has been observed in blood cells treated with Cat, CfA, and resveratrol [32]; in another study, GA inhibited CD production by 1,2-dimethyl hydrazine in rat colon cells [33]. Figure 6(a) also shows that treatment with CfA alone increased CD content to values close to H_2_O_2_-treated cells, which represents a prooxidant activity of this polyphenolic compound. This effect is in agreement with previous studies in which ferulic and caffeic acids showed prooxidant activity in cells at concentrations higher than 20 *μ*M but were antioxidant at lower concentrations. It is suggested that this change in activity can be associated with the capacity of hydroxycinnamic acids to reduce iron (Fe^+3^ to Fe^+2^) [34]. Treatment with CfA before or after H_2_O_2_ and with the other phenolic acids showed CD values similar to or lower than control.

TBARS values (Figure 6(b)) for all treatments with phenolic compounds were intermediate between control and H_2_O_2_-treated cells, except Cat alone and Cap in the presence or absence of H_2_O_2_, which were similar to control values. Considering that TBARS measures the secondary oxidation products, this indicates that all polyphenols were good antioxidants, but Cap was the best one. However, this result also shows that all phenolic acids and Qc have mild prooxidant effect when used in the absence of H_2_O_2_, in agreement with results obtained by the analysis of FTIRM and FTIR data. Protective effect of flavonoids against TBARS formation in tissues in response to different inductors of oxidative stress has been reported. Cat and resveratrol, at 75 *μ*M, reduced TBARS formation caused by *β*-amyloid peptide in PC-12 cells; and epigallocatechin-3-gallate, at 50 *μ*M, had the same effect in HepG2 cells stressed by CYP2E1 produced by ethanol stimuli [35, 36].

## 4. Concluding Remarks

All polyphenolic compounds showed a protective effect in rat enterocytes when administered before and after the oxidative stimulus (H_2_O_2_), when analyzed by FTIR spectra of single cells (FTIRM) or batch samples, and by classical biochemical methods for lipid oxidation (CD and TBARS). Cap and Cat showed the best protective effect. Under certain conditions, and in the absence of H_2_O_2_, all compounds behaved as mild prooxidants, but Qc and phenolic acids showed this behavior more consistently. The present results showed that analysis of FTIR and FTIRM spectra can provide valuable data on the effect of oxidant and antioxidant compounds in cellular systems because in a single and fast analysis one can evaluate effects on lipid and protein oxidation and the observed effects correlate with those observed by standard biochemical assays.

## Abbreviations

ATR: Attenuated Total Reflectance
CfA: Caffeic acid
Cat: Catechin
ChA: Chlorogenic acid
Cap: Capsaicin
FTIR: Fourier Transform Infrared Spectroscopy
FTIRM: Fourier Transform Infrared Microspectroscopy
GA: Gallic acid
MDA: Malondialdehyde
NIH: National Institutes of Health
PC: Principal Component
PCA: Principal Component Analysis
Qc: Quercetin
TBA: Thiobarbituric acid
TBARS: Thiobarbituric Acid Reactive Substances.
